# A temporal shift in trophic diversity among a predator assemblage in a warming Arctic

**DOI:** 10.1098/rsos.180259

**Published:** 2018-10-03

**Authors:** David J. Yurkowski, Nigel E. Hussey, Steven H. Ferguson, Aaron T. Fisk

**Affiliations:** 1 Department of Biological Sciences, University of Manitoba, Winnipeg, Manitoba, Canada R3T 2N2; 2 Department of Biological Sciences, University of Windsor, Windsor, Ontario, Canada N9B 3P4; 3 Freshwater Institute, Fisheries and Oceans Canada, Winnipeg, Manitoba, Canada R3T 2N6; 4 Great Lakes Institute for Environmental Research, University of Windsor, Windsor, Ontario, Canada N9B 3P4

**Keywords:** climate change, community-wide metrics, fishes, food web structure, marine mammals, stable isotopes

## Abstract

Climate change is leading to northward shifts in species distributions that is altering interspecific interactions at low- and mid-trophic levels. However, little attention has been focused on the effects of redistributions of species on the trophic ecology of a high trophic-level predator assemblage. Here, during a 22-year period (1990–2012) of increasing sea temperature (1.0°C) and decreasing sea ice extent (12%) in Cumberland Sound, Nunavut, Canada, we examined the trophic structure of a near-apex predator assemblage before (1990–2002) and after (2005–2012) an increase in the availability of capelin—generally an indicator species in colder marine environments for a warming climate. Stable isotopes (δ^13^C and δ^15^N) were used in a Bayesian framework to assess shifts in diet, niche size and community-wide metrics for beluga whales (*Delphinapterus leucas*), ringed seals (*Pusa hispida*), Greenland halibut (*Reinhardtius hippoglossoides*) and anadromous Arctic char (*Salvelinus alpinus*). After 2005, consumption of forage fish increased for all predator species, suggesting diet flexibility with changing abiotic and biotic conditions. An associated temporal shift from a trophically diverse to a trophically redundant predator assemblage occurred where predators now play similar trophic roles by consuming prey primarily from the pelagic energy pathway. Overall, these long-term ecological changes signify that trophic shifts of a high trophic-level predator assemblage associated with climate change have occurred in the Arctic food web.

## 1. Introduction

Biodiversity within marine ecosystems varies across environmental gradients, of which temperature is one of the most important and can regulate functioning of marine ecosystems [1]. With lower productivity at the poles when compared to lower latitudes, polar ecosystems are more consumer- than resource-controlled [2]. This consumer control leads to lower food web complexity and decreased connectance [3]. However, a warming climate is currently altering global ecosystem structure and driving species distributions to higher latitudes, thereby leading to altered interspecific interactions with unspecified consequences [4,5]. Climate-driven ecosystem shifts are pronounced in the Arctic—the fastest warming region on the planet [6]. Changes to Arctic sea temperature and sea ice phenology have facilitated a punctuated poleward shift in the distribution of more temperate species including apex predators (e.g. killer whales *Orcinus orca* [7]), near-apex predators (e.g. harp seals *Pagophilus groenlandicus* [8]), forage fish (e.g. capelin *Mallotus villosus* [9]) and invertebrates (e.g. blue mussels *Mytilus edulis* [10]). This has led to observed and ongoing changes to Arctic ecosystem productivity, species abundances, population mixing and disease/pathogen transmission for its fauna [11,12].

Forage fish are small pelagic species that provide the most important conduit of energy transfer from phytoplankton and zooplankton to predators in global marine ecosystems [13]. In the Arctic, endemic Arctic cod *Boreogadus saida* facilitate the majority of energy transfer (up to approx. 90%) to seabirds and marine mammals [14] but are probably undergoing an associated decline in abundance and shifts in distribution across several regions with a warming climate [3,15–17]. Arctic cod are typically associated with colder water temperatures (less than 5°C) [18], therefore declining sea ice and a warming ocean, especially at lower latitudes, will continue to alter the timing of Arctic cod reproduction and larval development and lead to a northward retraction in its range [16]. This retraction in range is exacerbated by the northward expansion of competitors, such as more temperate capelin that is also one of the most-used forage fish by marine mammals and sea birds in the Barents Sea and waters near Newfoundland [19,20]. Capelin are considered a sea ‘canary’ for a warming climate in colder marine ecosystems as their presence and abundance generally increase with temperature [9]. Furthermore, capelin can spawn over a large range of latitudes (42–72° N), temperatures (1—14°C) and habitats (e.g. beach and deep-water spawning), signifying their high plasticity to environmental variability and change [21,22].

Opportunistic upper trophic-level predators act as sentinels to trophodynamic and species assemblage changes lower in the food web through their diet [23]. For example, thick-billed murres (*Uria lomvia*) of Hudson Bay and polar bears (*Ursus maritimus*) of East Greenland have shifted their diet from Arctic cod to capelin, and from ringed seals (*Pusa hispida*) to subarctic seal species (e.g. hooded seals *Cystophora cristata*), respectively [24,25]. However, the effects of climate-driven dietary shifts on the overall community structure of concurrent near-apex predators are generally unknown. Here, we examine the diet and isotopic niche of beluga whales (*Delphinapterus leucas*), ringed seals, Greenland halibut (*Reinhardtius hippoglossoides*) and anadromous Arctic char (*Salvelinus alpinus*) across a temporal scale that captures a rapid warming period and where capelin have become increasingly abundant since the mid-2000s [19] (A Fisk 2006, personal observation and R Kilabuk from Pangnirtung, Nunavut, 2011, personal communication).

Beluga whales, ringed seals, Greenland halibut and Arctic char all inhabit Cumberland Sound (Nunavut, Canada) a large inlet where summer sea temperatures have increased by 1.0°C since 1990 (see Results). Beluga whales consume forage fish (Arctic cod [26] and increasingly capelin [27,28]), Greenland halibut [29], squid and benthic invertebrates (decapods and amphipods [26,30]). Ringed seal and Greenland halibut diet consists of a wide variety of pelagic invertebrates including *Gonatid* squid [31,32] and forage fish (e.g. Arctic cod, capelin and sand lance [33–35]), while Arctic char consume invertebrates (e.g. amphipods and shrimp) and forage fish (e.g. herring and capelin [36]).

Stable isotope analysis of animal tissues provides time-integrated information on habitat use and diet and has become one of the principal tools to elucidate prey contributions to predator diet and spatio-temporal variation of trophic interactions among species [37]. Specifically, combined δ^13^C and δ^15^N data have been used to quantify inter-annual variation and long-term changes in trophic structure of aquatic systems [38–40]. Stable isotopes consequently provide a proven tool to assess the impact of human-driven climate shifts on the structure and dynamics of predator communities in polar aquatic environments [37,41].

Here, we determine prey contributions to the diet of beluga whales, ringed seals, Greenland halibut and Arctic char, quantify predator niche sizes and apply six community-wide metrics to characterize the trophic structure of this near apex predator assemblage in Cumberland Sound, Nunavut, Canada (65°13′0″ N, 65°45′0″ W). We provide the first empirical evidence of long-term (1990–2012) alteration to the trophic structure of a near-apex predator assemblage associated with changes in the composition of forage fish species availability coincident with a rapidly warming climate.

## 2. Material and methods

### 2.1. Environmental data

Mean summer sea surface temperatures in Cumberland Sound for the study period 1990–2012 (June–October) were obtained from National Oceanographic and Atmospheric Administration, Earth System Research Laboratory (http://www.esrl.noaa.gov/psd/data/gridded/data.noaa.oisst.v2.html, accessed September 2016) at 1° latitude × 1° longitude spatial resolution. Sea ice concentration for the Davis Strait was also estimated for the same period for the month of June—a month which mainly encompasses sea ice breakup in the area—using Canadian Ice Service's IceGraph 2.0 Tool (http://iceweb1.cis.ec.gc.ca/IceGraph, accessed September 2016).

### 2.2. Sample collections

To allow examination of a potential shift in predator diet, we separated all predator and prey sample collections into two time periods (1990–2002 and 2005–2012) to coincide with increased availability of capelin in Cumberland Sound (mid-2000s [19]). Inuit hunters inhabiting Southeast Baffin Island have not reported the occurrence of capelin in beluga whale stomachs in the 1990s despite their opportunistic feeding strategy [29], thereby supporting a recent shift in capelin availability. Division of the sampling period into these two time periods was further defined by a significant decrease in beluga whale δ^15^N and sympagic carbon source use for both beluga whales and ringed seals after the early 2000s in Cumberland Sound [42,43]. Beluga whale and ringed seal muscle samples were collected during May–October by Inuit hunters in Cumberland Sound as part of their subsistence harvests during 1992–2009 and 1990–2011, respectively (table 1; see electronic supplementary material, table S1 for sample size by year per species). The beluga whale population inhabits Cumberland Sound year-round [44] and Cumberland Sound ringed seal movements are generally restricted during the summer (D Yurkowski 2011, unpublished data). Greenland halibut were captured from bottom longlines in western Davis Strait near the entrance to Cumberland Sound in September 1996 and again during August 2012 in the central region of Cumberland Sound. Arctic char were collected from gill nets set from shore at tidal flats in 2002, 2008 and 2011 in northern Cumberland Sound near Lake Kipisa and Isuituq, and stable isotope values were obtained from [45]. Year-round movements of Greenland halibut occur in Cumberland Sound [46] and at-sea movements of anadromous Arctic char are generally restricted [47]. Muscle samples from all predator species represent long-term dietary integration of prey due to its slower turnover rate compared to more metabolically active tissues [48]. Stable isotope values of shrimp (*Pandalus borealis*) and Arctic cod were obtained from [49] which were caught from fishing vessel trawls in western Davis Strait near the entrance to Cumberland Sound in October 2000, 2001 and 2004. Arctic cod collected in 2004 were categorized in the 2005–2012 time period. Shrimp (*Lebbeus polaris*) and capelin samples were collected from Cumberland Sound in August 2007–2009. Owing to the unavailability of *Themisto* sp., a pelagic omnivorous invertebrate and common prey item for Arctic marine predators, *Gonatid* squid stable isotope values (caught September–October in 2001 and 2011) were used instead to represent this functional group (e.g. omnivorous invertebrate [50]). All predator and prey sample tissues were stored at −20°C prior to analysis.

**Table 1.** Summary of δ^13^C and δ^15^N (mean ± s.d.), δ^13^C and δ^15^N ranges and median Bayesian standard ellipse area (SEA_B_) by time period for predator species from Cumberland Sound, Nunavut, Canada.

common name	*n*	δ^13^C (‰)	δ^15^N (‰)	δ^13^C range (‰)	δ^15^N range (‰)	SEA_B_ (‰^2^)
1990–2002						
beluga	47	−18.1 ± 0.3	17.2 ± 1.1	1.5	5.2	1.0
ringed seal	175	−18.7 ± 0.6	15.2 ± 0.9	3.3	4.8	1.7
Greenland halibut	14	−19.6 ± 0.7	16.6 ± 0.4	2.3	1.4	0.8
Arctic char	72	−20.0 ± 0.5	15.0 ± 0.7	2.2	2.7	0.9
2005–2012						
beluga	25	−18.3 ± 0.4	15.9 ± 0.8	1.3	3.0	1.0
ringed seal	53	−19.1 ± 0.5	15.0 ± 0.8	2.2	3.2	1.3
Greenland halibut	21	−19.4 ± 0.4	16.4 ± 0.7	1.7	2.5	0.7
Arctic char	122	−19.1 ± 0.6	15.0 ± 0.7	3.3	3.6	1.3

### 2.3. Stable isotope analysis

Owing to the presence of lipids affecting fish and mammal tissue δ^13^C values [51,52], frozen predator and prey samples were lyophilized for 48 h, homogenized using a mortar and pestle and lipid-extracted using 2 : 1 chloroform : methanol following the methods of [53]. Subsequently, 400–600 µg of predator and prey tissue were weighed into tin capsules and δ^13^C and δ^15^N values measured by a Thermo Finnigan DeltaPlus mass spectrometer (Thermo Finnigan, San Jose, CA, USA) coupled with an elemental analyser (Costech, Valencia, CA, USA) at the Chemical Tracers Laboratory, Great Lakes Institute for Environmental Research, University of Windsor. Stable isotope ratios are expressed in per mil (‰) in delta (δ) notation using the following equation: δX = [(*R*sample/*R*standard) − 1] × 10^3^, where X is ^13^C or ^15^N and *R* equals ^13^C/^12^C or ^15^N/^14^N. The standard reference material was Pee Dee Belemnite carbonate for CO_2_ and atmospheric nitrogen N_2_. A triplicate was run for every 10th sample, and a measurement precision for δ^13^C and δ^15^N was 0.1‰ and 0.1‰, respectively. The instrumentation accuracy was determined based on NIST standards 8573, 8547 and 8548 for δ^15^N values and 8542, 8573, 8574 for δ^13^C values (*n* = 75 for all). The mean differences from the certified values were ≤0.1*‰* for δ^15^N values and ≤0.1*‰* for δ^13^C values.

### 2.4. Data analysis

To examine shifts in environmental parameters over the study period, linear regression of sea surface temperatures and logit-transformed sea ice concentration versus year (1990–2012) were performed. Alpha was set to 0.05. To quantify prey contributions to focal predators (beluga whale, ringed seal, Greenland halibut and Arctic char) over the two defined study periods, we used Bayesian mixing model analysis in SIAR v. 4.2.2 [54] in R v. 3.3.2 [55] with uninformative priors. These mixing models were run at 500 000 iterations, a burn-in of 300 000 and thinned by 100 (see table 2 for δ^13^C and δ^15^N values of each prey and electronic supplementary material, figures S1 and S2 for stable isotope bi-plots). Prior to analysis, we assessed normality of δ^13^C and δ^15^N using a χ^2^ quantile–quantile plot for each predator species. As capelin and Arctic cod are a part of the same functional group (i.e. forage fish) and δ^13^C and δ^15^N values for the two species were similar (capelin: mean ± s.d.; −19.5‰ ± 0.3 and 13.8‰ ± 0.5; Arctic cod: −20.4‰ ± 0.5 and 13.7‰ ± 1.1, respectively), these prey items were combined to reduce the total number of prey sources to four and allow a more constrained, diffuse solution [56]. For ringed seals and beluga whales, we used known diet tissue discrimination factors (DTDF) for phocid muscle (Δ^13^C: 1.3‰, Δ^15^N: 2.4‰ [57]) and cetacean muscle (Δ^13^C: 1.3‰, Δ^15^N: 1.2 ‰ [58]), respectively. We estimated DTDFs for Greenland halibut and Arctic char muscle using linear models from meta-analysis of fish muscle δ^13^C and δ^15^N values relative to diet isotope values; 1.2‰ for Δ^13^C and 2.1‰ for Δ^15^N [59,60]. Following the recommendation of [54], we incorporated variability in DTDFs (standard deviation = 0.2‰ for δ^13^C and δ^15^N) for each species (see the electronic supplementary material for other practical assumptions). As well, few individuals who did not fall within simulated mixing polygons (i.e. statistical outliers) were removed prior to mixing model analysis (see [61]; electronic supplementary material, figures S3 and S4). We estimated the probability that contributions of forage fish to each predator diet were higher in 2005–2012 than 1990–2002 by calculating the percentage of estimates from the posterior probability distribution that were higher in 2005–2012 than 1990–2002 relative to the total number of estimates from the posterior probability distribution (2000).

**Table 2.** Mean ± s.d. of δ^13^C (‰) and δ^15^N (‰) values for potential prey items and their median contribution (95% Bayesian credible interval) to predator diet by time period from Cumberland Sound, Nunavut, Canada.

common name	species name	*n*	δ^13^C (‰)	δ^15^N (‰)	contribution to beluga diet (%)	contribution to ringed seal diet (%)	contribution to Greenland halibut diet (%)	contribution to Arctic char diet (%)
1990–2002								
squid	*Gonatid* sp*.*	7	−20.3 ± 0.9	11.4 ± 0.9	1 (0–4)	64 (60–68)	6 (0–18)	59 (47–71)
shrimp^b^	*Pandalus borealis*	10	−18.7 ± 0.5	13.3 ± 1.0	19 (3–33)	6 (0–14)	13 (0–38)	11 (0–33)
Arctic cod^b^	*Boreogadus saida*	8	−19.2 ± 0.5	14.1 ± 1.2	15 (1–36)	12 (1–23)	79 (55–97)	29 (10–44)
Greenland halibut	*Reinhardtius hippoglossoides*	14	−19.6 ± 0.7	16.6 ± 0.4	63 (51–74)	19 (13–24)	—	—
2005–2012								
squid	*Gonatid* sp*.*	5	−19.8 ± 0.6	11.2 ± 1.3	14 (0–31)	48 (39–58)	2 (0–8)	34 (29–39)
shrimp^a^	*Lebbeus polaris*	7	−18.2 ± 0.2	13.9 ± 0.4	8 (0–18)	1 (0–4)	2 (0–10)	1 (0–2)
Arctic cod/capelin^a^	*Boreogadus saida/ Mallotus villosus*	22	−20.0 ± 0.4	13.7 ± 0.8	35 (3–56)	49 (34–60)	95 (87–100)	66 (61–71)
Greenland halibut	*Reinhardtius hippoglossoides*	21	−19.4 ± 0.4	16.4 ± 0.7	42 (27–63)	2 (0–9)	—	—

^a^Source [30].

^b^Source [49].

To examine variation in isotopic niche sizes and relative niche position for each predator species over the two study time periods, standard ellipses were estimated using the SIBER package v. 2.0.3 [62] in R which also uses Bayesian inference. Each ellipse represents the variance and covariance of *x* and *y* thereby containing approximately 40% of the total data [62]. The niche ranges measured by the highest and lowest individual δ^13^C and δ^15^N values were calculated for each species separately. This allowed the ability to discern which predator species contributed more to the temporal changes in isotope values of the predator assemblage. We estimated the Bayesian standard ellipse area (SEA_B_: iterations = 2 000 000, burn-in = 100 000, thin by = 10) for statistical comparisons among predators. To examine differences in SEA_B_ between both time periods, we calculated percentage of estimates from the posterior probability distribution for SEA_B_ that were lower in 2005–2012 versus 1990–2002 relative to the total number of estimates from the posterior probability distribution (10 000).

Six community-wide metrics representative of the interactions among the realized niches of the four-predator species assemblage were also estimated using SIBER with Bayesian inference. These metrics include total extent of spacing within δ^13^C–δ^15^N bi-plot space and the relative trophic position of the predator assemblage to provide a measure of trophic diversity and redundancy. The δ^13^C and δ^15^N ranges measure the distance between the two individuals with highest and lowest values, and thus represent the variability in basal carbon source and relative trophic position of the predator assemblage. Mean distance to centroid is the mean Euclidean distance of each species' niche to the δ^13^C–δ^15^N centroid of the predator assemblage and represents the overall degree of trophic diversity. Mean nearest neighbour distance is the mean Euclidean distance to each species’ nearest neighbour in isotopic space, thereby representing density of species packing where species with similar trophic ecologies (i.e. trophic redundancy) exhibit smaller mean nearest neighbour distances. Standard deviation of the nearest neighbour distance is the standard deviation of Euclidean distance of each species to its nearest neighbour and thus represents packing of species in isotopic space and trophic redundancy. Total community area was modified by calculating the total isotopic area among the means of each species' niche and thus is less biased to convex hull extremities [63]. Total community area is used as a proxy for the total extent of trophic diversity within the predator assemblage. All six metrics were derived from 2 000 000 iterations, a burn-in of 100 000 and thinned by 10 leaving 10 000 posterior estimates from the posterior probability distribution. To determine differences in community-wide metrics between both time periods, we quantified the percentage of estimates from the posterior probability distribution that were lower in 2005–2012 versus 1990–2002 relative to the total number of estimates from the posterior probability distribution (10 000). The ranges of δ^13^C and δ^15^N of selected prey sources between both time periods were similar allowing a comparison of the community-wide metrics between both time periods (table 2). To eliminate bias associated with DTDF variability by taxa, body size and diet [59], all focal predator isotope values were corrected with designated DTDFs prior to trophic structure analysis. In regard to the division of sampling periods (1990–2002 and 2005–2012) and increased capelin availability in the mid-2000s, similar results of analyses described above from 2007–2012 are provided in the electronic supplementary material.

## 3. Results

Over the entire study period (1990–2012), there were marked shifts in sea ice concentrations in the Davis Strait and summer sea surface temperatures within Cumberland Sound; a 12% decline in sea ice extent (slope = −0.025, intercept = 49.30, *r*^2^ = 0.23, *p* = 0.02) occurred and an increase in temperature of 1°C was found (slope = 0.043, intercept = −85.50, *r*^2^ = 0.24, *p* < 0.001).

**Figure 1.**
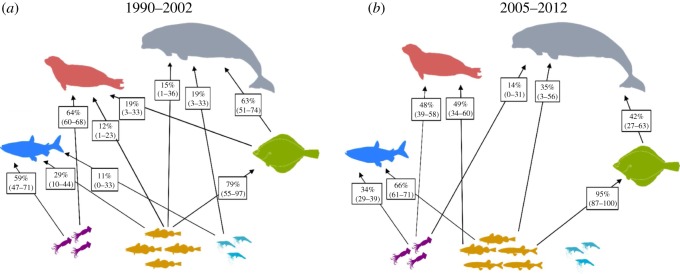
Stable isotope mixing model results depicting the median contributions (95% Bayesian credible intervals) of prey ≥ 10% to beluga, ringed seal, Greenland halibut and Arctic char diet from 1990–2002 to 2005–2012 from Cumberland Sound, Nunavut, Canada. Species symbols represent beluga (grey), ringed seals (pink), Greenland halibut (green), Arctic char (blue) squid (purple), shrimp (blue) and forage fish (Arctic cod during 1990–2002 and Arctic cod/capelin during 2005–2012; yellow).

A comparison of the posterior distributions of prey items from stable isotope mixing models revealed that Cumberland Sound beluga whale diet consisted predominantly of Greenland halibut during both focal time periods, but there was a 94% probability that its contribution to diet decreased between 1990–2002 and 2005–2012 (table 2 and figure 1). Concomitantly, the probability of increased forage fish in the diet of beluga whales between 1990–2002 and 2005–2012 was 81% (table 2 and figure 1). Ringed seal diet consisted of both squid and forage fish, but similar to beluga whales, a probability of increased consumption of forage fish was greater than 99% from 1990–2002 to 2005–2012. In turn, the probability of a decreased squid contribution to ringed seal diet between the two time periods was greater than 99% (table 2 and figure 1). The probability of an increased forage fish contribution to Greenland halibut diet between 1990–2002 and 2005–2012 was 97% where their diet principally consisted of forage fish (95%; Bayesian credible intervals: 87–100; table 2 and figure 1). For Arctic char, the probability of increased consumption of forage fish in their diet from 1990–2002 to 2005–2012 was greater than 99% (table 2 and figure 1).

**Figure 2.**
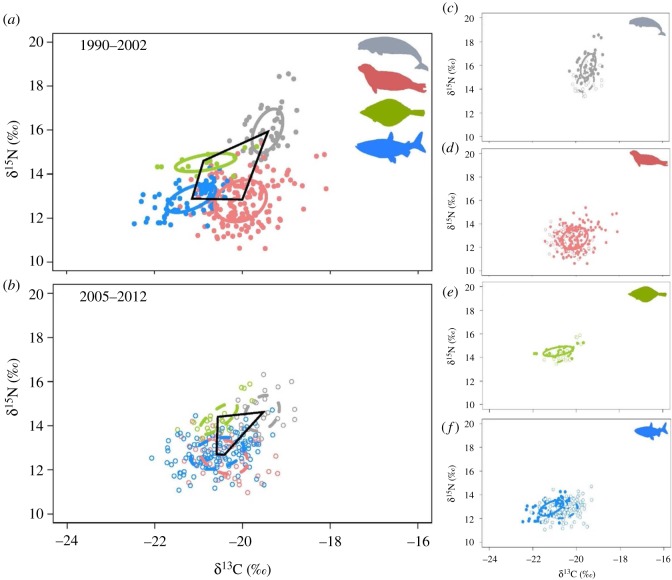
Stable isotope bi-plot representing the 40% isotopic niche sizes of beluga, ringed seals, Greenland halibut and Arctic char during 1990–2002 (solid lines; (*a*)) and 2005–2012 (dashed lines; (*b*)) with solid black lines characterizing the community metric of total area. (*c–f*) Isotopic niche shifts for each predator species between both time periods. Symbols and ellipses are colour-coded by species similar to figure 1.

The probability that SEA_B_ size for ringed seals became smaller between the two time periods was 95%, whereas the probability that SEA_B_ size for Arctic char increased over time was 99%. By species, isotopic niche shifts occurred along the δ^13^C-axis for Greenland halibut and Arctic char and along the δ^15^N-axis for beluga whales (figure 2). Furthermore, the δ^13^C range of Greenland halibut and ringed seals decreased by 0.6 and 1.1‰, respectively, between 1990–2002 and 2005–2012, while the δ^15^N range increased by 1.1‰ for Greenland halibut, and decreased by 1.6 and 1.1‰ for ringed seals and beluga whales, respectively (table 1). For Arctic char, the δ^13^C and δ^15^N ranges increased by 1.1 and 0.9‰, respectively, between 1990–2002 and 2005–2012 (table 1).

### 3.1. Temporal changes in community structure of an Arctic predator assemblage

**Figure 3.**
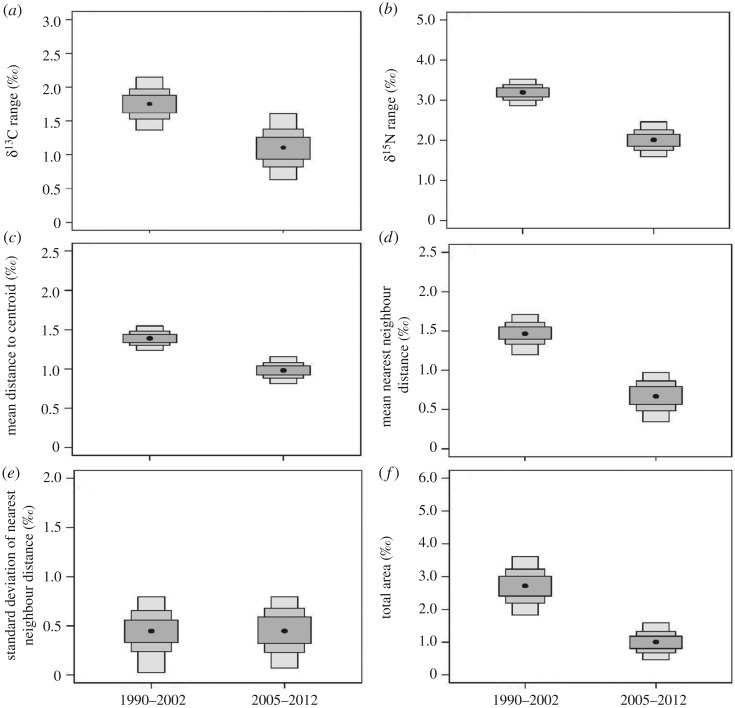
Boxplots representing Bayesian mode estimates for each community-wide metric including δ^13^C range (*a*), δ^15^N range (*b*), mean distance to centroid (*c*), mean nearest neighbour distance (*d*), standard deviation of nearest neighbour distance (*e*) and total area (*f*) from 1990–2002 to 2005–2012. Boxes indicate Bayesian credible intervals at 50% (dark grey), 75% (medium grey) and 95% (light grey).

All six community-wide metrics of trophic structure were lower in the most recent sampling period (2005–2012) than the 1990–2002 time period (figure 3). By analysing the posterior distribution, the probability of lower δ^13^C (i.e. variability in basal carbon source use) and δ^15^N (i.e. relative trophic position) ranges for the predator assemblage in 2005–2012 was greater than 98% (figures 2 and 3). The probability of mean distance to centroid, mean nearest neighbour distance and total area being lower in 2005–2012 compared to 1990–2002 were all greater than 99%, identifying a decrease in trophic diversity and higher trophic redundancy among the predator assemblage over time (figures 2 and 3). The probability of a decrease in the standard deviation of nearest neighbour distance in 2005–2012 was 50% (figures 2 and 3).

## 4. Discussion

With a warming ocean and continuing reduction in sea ice extent, Arctic marine ecosystems continue to face multiple abiotic and biotic stressors that are impacting species interactions and overall ecosystem structure and function [11,64]. While temporal shifts in diet related to climate have been documented for several endemic upper trophic-level predators in the Arctic [24,25], our study provides evidence for a simultaneous isotopic niche shift of several sympatric higher trophic position predators over the past two decades. The observed predator assemblage shifts are associated with interactions among abiotic and biotic variables including decreased sea ice concentration, increased summer sea surface temperature and changes to forage fish species composition in Cumberland Sound over the 22-year period. Our stable isotope mixing model estimates, which represent the proportional contributions of prey to predator diets, demonstrate that the predator assemblage now consumes more pelagic forage fish during 2005–2012 than 1990–2002 probably through increased capelin availability, an expanding species from the south. These temporal shifts in diet of the predator assemblage indicate flexibility in foraging tactics of Arctic marine predators in response to abiotic and biotic change. Moreover, these data provide strong support of a temporal shift from a trophically diverse to a more trophically redundant predator assemblage associated with climate change.

Beluga whales, ringed seals, Greenland halibut and Arctic char exhibited flexible foraging behaviour over the study period with consumption of higher proportions of forage fish in the 2005–2012 time period. Flexible foraging behaviour is pervasive in nature and allows opportunistic predators to exploit shifting prey diversity and abundance in response to seasonal and inter-annual variations in environment [65]. For example, a substantial increase in the consumption of capelin associated with a decline in sea ice and decreased consumption of sympagic Arctic cod in diets of thick-billed murres over time was, at least in part, due to increased availability of capelin modulating a switch to this prey species [24].

The most probable explanation for the increase of forage fish consumption among the four near-apex predator species is the increased availability of capelin since the mid-2000s. Cumberland Sound beluga whales have been previously documented to consume both Arctic cod and capelin, though with an increasing reliance on capelin over time [27,30]. In addition, Cumberland Sound beluga whales dive to shallower depths (0–100 m) in the summer compared to the late-autumn and winter where dives are greater than 400 m, suggesting a seasonal switch from foraging on forage fish in shallower waters to deep-water fishes such as Greenland halibut [27,66]. Ringed seals have been shown to respond to varying prey availability and distribution by exhibiting high flexibility in their movement ecology and diet with increasing latitude [35,67]. With decreasing sea ice extent, Cumberland Sound ringed seals have also been shown to be less dependent on sympagic carbon and more dependent on pelagic carbon [42], further supporting our results of a substantial increase in consumption of forage fish over time. Greenland halibut have been reported to consume Arctic cod during the open water period at higher latitudes [68] and capelin in Cumberland Sound [69] which is also consistent with our mixing model results. For Arctic char, stomach content analysis of individuals in Cumberland Sound found a diet switch from invertebrates to a capelin-dominated diet in the late 2000s [45], comparable to our mixing model results. Arctic char adopts an opportunistic foraging strategy where their diet typically reflects prey availability, especially when exploiting high density prey patches [70], such as large aggregations of beach-spawning capelin. Therefore, increased capelin availability allows greater accessibility for Arctic char to consume a high-lipid prey item which, in turn, positively affects growth rates [45] and body condition [71].

Given that capelin are highly adapted to survive across a broad range of temperatures (2°C to 12°C), and their northward expansion in distribution is linked with increasing sea temperatures [9], Cumberland Sound provides ideal colonizing potential for this highly plastic species. Beach spawning of capelin is now prevalent in Cumberland Sound during the summer and there are indications of capelin overwintering (see electronic supplementary material, figure S5), thereby increasing its overall availability to predators throughout the year. Scant details are known about capelin's overall occurrence and distribution across the Arctic, as most current information comes from either Inuit observation or studies on temporal changes in seabird diet [19,24]. However, distributional shifts of the northwest and northeast-central Atlantic capelin related to temperature have occurred recently in waters around Newfoundland, Labrador and Iceland, and a northeastward shift has been reported in the Barents Sea [19]. With high capacity to track suitable climatic conditions, these northward distributional shifts of more-temperate fish species such as capelin to warming Arctic waters are predicted to accelerate [72].

Comparison of the metrics measuring the trophic structure of the predator assemblage between 1990–2002 and 2005–2012 in Cumberland Sound revealed the latter having less variability in basal carbon source use and trophic position, such that the community exhibited less trophic diversity and more trophic redundancy. Warming waters and phenological changes to sea ice over time strongly influence the seasonal pulse of algal productivity during the summer allowing more prolonged availability of this energy pathway to consumers [64]. In turn, the sympagic and detrital energy pathways probably become dampened leading to predators and prey capitalizing on resources from the pelagic energy pathway, thereby decreasing overall variability in basal carbon source use among the predator assemblage. Furthermore, lower variability in the relative trophic position of the predator community in 2005–2012 was predominantly driven by a niche shift in beluga whale diet from consuming less Greenland halibut to a higher proportion of forage fish. Cumberland Sound trophic diversity (i.e. mean distance to centroid and total area) decreased, while trophic redundancy (i.e. mean nearest neighbour distance) increased over time. This suggests that the individual species of the predator assemblage now play similar trophic roles within the food web by primarily consuming resources from the pelagic energy pathway and occupying a more-similar trophic position.

Climate-driven community shifts in the Arctic will probably accelerate with warming temperatures and decreasing sea ice leading to more subarctic and temperate species, from phytoplankton to predators, invading Arctic waters [8]. This northward shift will probably have detrimental consequences on Arctic community composition by causing a decline in the abundance of endemic Arctic species with associated consequences on the functional biogeography and spatial coupling between pelagic and benthic energy compartments of the ecosystem [19,73]. For example, functional traits typical of boreal marine fish communities (e.g. larger body sizes, increased piscivory and utilization of pelagic resources and high generalism) are becoming more prevalent in the Barents Sea and impacting its trophic structure and dynamics [3,73]. Over a 22-year period, our study in Cumberland Sound found dietary shifts among a sympatric predator assemblage resulting in decreased trophic diversity and increased trophic redundancy with potential implications on spatial coupling between benthic and pelagic energy compartments. Given the scale of defaunation in the current Anthropocene [74] and marine taxa tracking climate velocities by expanding or retracting their ranges [75], the reconfiguration of ecological interactions in the Arctic will persist and probably intensify in the future leading to continued change in the structure, function and resilience of Arctic food webs.

## Supplementary Material

Electronic Supplementary Material 1

## Supplementary Material

Electronic Supplementary Material - Data

## Data accessibility

Data supporting our results are provided in the electronic supplementary material.

## References

[1] Tittensor DP, Mora C, Jetz W, Lotze HK, Ricard D, Berghe EV, Worm B 2010 Global patterns and predictors of marine biodiversity across taxa. Nature 466, 1098–1101. (10.1038/nature09329) 20668450

[2] Boyce DG, Frank KT, Worm B, Leggett WC 2015 Spatial patterns and predictors of trophic control in marine ecosystems. Ecol. Lett. 18, 1001–1011. (10.1111/ele.12481) 26252155

[3] Kortsch S, Primicerio R, Fossheim M, Dolgov AV, Aschan M 2015 Climate change alters the structure of arctic marine food webs due to poleward shifts of boreal generalists. Proc. R. Soc. B 282, 20151546 (10.1098/rspb.2015.1546) PMC4571709 26336179

[4] Walther GR, Post E, Convey P, Menzel A, Parmesan C, Beebee TJ, Fromentin JM, Hoegh-Guldberg O, Bairlein F 2002 Ecological responses to recent climate change. Nature 416, 389–395. (10.1038/416389a) 11919621

[5] Parmesan C, Yohe G 2003 A globally coherent fingerprint of climate change impacts across natural systems. Nature 421, 37–42. (10.1038/nature01286) 12511946

[6] Intergovernmental Panel on Climate Change. 2013 Climate Change 2013: The Physical Science Basis. Working Group II Contribution to the IPCC 5th Assessment Report.

[7] Higdon JW, Ferguson SH 2009 Loss of Arctic sea ice causing punctuated change in sightings of killer whales (*Orcinus orca*) over the past century. Ecol. Appl. 19, 1365–1375. (10.1890/07-1941.1) 19688941

[8] Laidre KL, Stirling I, Lowry LF, Wiig Ø, Heide-Jørgensen MP, Ferguson SH 2008 Quantifying the sensitivity of Arctic marine mammals to climate-induced habitat change. Ecol. Appl. 18, S97–S125. (10.1890/06-0546.1) 18494365

[9] Rose GA 2005 Capelin (*Mallotus villosus*) distribution and climate: a sea ‘canary’ for marine ecosystem change. ICES J. Mar. Sci. 62, 1524–1530. (10.1016/j.icesjms.2005.05.008)

[10] Berge J, Johnsen G, Nilsen F, Gulliksen B, Slagstad D 2005 Ocean temperature oscillations enable reappearance of blue mussels *Mytilus edulis* in Svalbard after a 1000 year absence. Mar. Ecol. Prog. Ser. 303, 167–175. (10.3354/meps303167)

[11] Wassmann P, Duarte CM, Agusti S, Sejr MK 2011 Footprints of climate change in the Arctic marine ecosystem. Global Change Biol. 17, 1235–1249. (10.1111/j.1365-2486.2010.02311.x)

[12] Descamps S, Aars J, Fuglei E, Kovacs KM, Lydersen C, Pavlova O, Pedersen ÅØ, Ravolainen V, Strøm H 2017 Climate change impacts on wildlife in a High Arctic archipelago–Svalbard, Norway. Global Change Biol. 23, 490–502. (10.1111/gcb.13381) 27250039

[13] Pikitch EK et al. 2014 The global contribution of forage fish to marine fisheries and ecosystems. Fish Fish. 15, 43–64. (10.1111/faf.12004)

[14] Welch HE, Crawford RE, Hop H 1993 Occurrence of Arctic cod (*Boreogadus saida*) schools and their vulnerability to predation in the Canadian High Arctic. Arctic 46, 331–339. (doi:10.14430/arctic1361)

[15] Gaston AJ, Woo K, Hipfner JM 2003 Trends in forage fish populations in northern Hudson Bay since 1981, as determined from the diet of nestling thick-billed murres *Uria lomvia*. Arctic 56, 227–233. (doi:10.14430/arctic618)

[16] Hop H, Gjøsæter H 2013 Polar cod (*Boreogadus saida*) and capelin (*Mallotus villosus*) as key species in marine food webs of the Arctic and the Barents Sea. Mar. Biol. Res. 9, 878–894. (10.1080/17451000.2013.775458)

[17] Vihtakari M, Welcker J, Moe B, Chastel O, Tartu S, Hop H, Bech C, Descamps S, Gabrielsen GW 2018 Black-legged kittiwakes as messengers of Atlantification in the Arctic. Sci. Rep. 8, 1178 (10.1038/s41598-017-19118-8) 29352216 PMC5775339

[18] Laurel BJ, Spencer M, Iseri P, Copeman LA 2016 Temperature-dependent growth and behaviour of juvenile Arctic cod (*Boreogadus saida*) and co-occurring North Pacific gadids. Pol. Biol. 39, 1127–1135. (10.1007/s00300-015-1761-5)

[19] Carscadden JE, Gjøsæter H, Vilhjálmsson H 2013 A comparison of recent changes in distribution of capelin (*Mallotus villosus*) in the Barents Sea, around Iceland and in the Northwest Atlantic. Prog. Oceanogr. 114, 64–83. (10.1016/j.pocean.2013.05.005)

[20] Fossheim M, Primicerio R, Johannesen E, Ingvaldsen RB, Aschan MM, Dolgov AV 2015 Recent warming leads to a rapid borealization of fish communities in the Arctic. Nat. Clim. Change 5, 673–677. (10.1038/nclimate2647)

[21] Stergiou KI 1989 Capelin *Mallotus villosus* (Pisces: Osmeridae), glaciations, and speciation: a nomothetic approach to fisheries ecology and reproductive biology. Mar. Ecol. Prog. Ser. 56, 211–224. (10.3354/meps056211)

[22] Carscadden J, Nakashima BS, Frank KT 1997 Effects of fish length and temperature on the timing of peak spawning in capelin (*Mallotus villosus*). Can. J. Fish. Aquat. Sci. 54, 781–787. (10.1139/f96-331)

[23] Moore SE, Huntington HP 2008 Arctic marine mammals and climate change: impacts and resilience. Ecol. Appl. 18, S157–S165. (10.1890/06-0571.1) 18494369

[24] Provencher JF, Gaston AJ, Hara PO, Gilchrist HG 2012 Seabird diet indicates changing Arctic marine communities in eastern Canada. Mar. Ecol. Prog. Ser. 454, 171–182. (10.3354/meps09299)

[25] McKinney MA et al. 2013 Global change effects on the long-term feeding ecology and contaminant exposures of East Greenland polar bears. Global Change Biol. 19, 2360–2372. (10.1111/gcb.12241) 23640921

[26] Loseto LL, Stern GA, Connelly TL, Deibel D, Gemmill B, Prokopowicz A, Fortier L, Ferguson SH 2009 Summer diet of beluga whales inferred by fatty acid analysis of the eastern Beaufort Sea food web. J. Exp. Mar. Biol. Ecol. 374, 12–18. (10.1016/j.jembe.2009.03.015)

[27] Watt CA, Orr J, Ferguson SH 2016 A shift in foraging behaviour of beluga whales *Delphinapterus leucas* from the threatened Cumberland Sound population may reflect a changing Arctic food web. Endangered Species Res. 31, 259–270. (10.3354/esr00768)

[28] Kelley TC, Loseto LL, Stewart RE, Yurkowski M, Ferguson SH 2010 Importance of eating capelin: unique dietary habits of Hudson Bay beluga. In A little less Arctic, pp. 53–70. Amsterdam, The Netherlands: Springer.

[29] Kilibuk P. 1998 A study of Inuit knowledge of southeast Baffin beluga, pp. 1–82. Iqaluit, NU: Nunavut Wildlife Management Board.

[30] Marcoux M, McMeans BC, Fisk AT, Ferguson SH 2012 Composition and temporal variation in the diet of beluga whales, derived from stable isotopes. Mar. Ecol. Prog. Ser. 471, 283–291. (10.3354/meps10029)

[31] Lowry LF, Frost KJ, Burns JJ 1980 Variability in the diet of ringed seals, *Phoca hispida*, in Alaska. Can. J. Fish. Aquat. Sci. 37, 2254–2261. (10.1139/f80-270)

[32] Bowering WR, Lilly GR 1982 Greenland halibut (*Reinhardtius hippoglossoides*) off southern Labrador and northeastern Newfoundland (Northwest Atlantic) feed primarily on capelin (*Mallotus villosus*). Netherlands J. Sea Res. 29, 211–222. (10.1016/0077-7579(92)90021-6)

[33] Dwyer KS, Buren A, Koen-Alonso M 2010 Greenland halibut diet in the Northwest Atlantic from 1978 to 2003 as an indicator of ecosystem change. J. Sea Res. 64, 436–445. (10.1016/j.seares.2010.04.006)

[34] Chambellant M, Stirling I, Ferguson SH 2013 Temporal variation in western Hudson Bay ringed seal *Phoca hispida* diet in relation to environment. Mar. Ecol. Prog. Ser. 481, 269–287. (10.3354/meps10134)

[35] Yurkowski DJ, Ferguson SH, Semeniuk CA, Brown TM, Muir DC, Fisk AT 2016 Spatial and temporal variation of an ice-adapted predator's feeding ecology in a changing Arctic marine ecosystem. Oecologia 180, 631–644. (10.1007/s00442-015-3384-5) 26210748

[36] Dempson JB, Shears M, Bloom M 2002 Spatial and temporal variability in the diet of anadromous Arctic char, *Salvelinus alpinus*, in northern Labrador. In Ecology, behaviour and conservation of the chars, genus Salvelinus, pp. 49–62. Amsterdam, The Netherlands: Springer.

[37] Layman CA et al. 2012 Applying stable isotopes to examine food-web structure: an overview of analytical tools. Biol. Rev. 87, 545–562. (10.1111/j.1469-185X.2011.00208.x) 22051097

[38] Jennings S, Greenstreet SPR, Hill L, Piet GJ, Pinnegar JK, Warr KJ 2002 Long-term trends in the trophic structure of the North Sea fish community: evidence from stable-isotope analysis, size-spectra and community metrics. Mar. Biol. 141, 1085–1097. (10.1007/s00227-002-0905-7)

[39] Jackson MC, Donohue I, Jackson AL, Britton JR, Harper DM, Grey J 2012 Population-level metrics of trophic structure based on stable isotopes and their application to invasion ecology. PLoS ONE 7, e31757 (10.1371/journal.pone.0031757) 22363724 PMC3283663

[40] Schmidt SN, Vander Zanden MJ, Kitchell JF 2009 Long-term food web change in Lake Superior. Can. J. Fish. Aquat. Sci. 66, 2118–2129. (10.1139/F09-151)

[41] Moreno R, Stowasser G, McGill RAR, Bearhop S, Phillips RA 2016 Assessing the structure and temporal dynamics of seabird communities: the challenge of capturing marine ecosystem complexity. J. Anim. Ecol. 85, 199–212. (10.1111/1365-2656.12434) 26439671 PMC4989482

[42] Brown TA, Alexander C, Yurkowski DJ, Ferguson SH, Belt ST 2014 Identifying variable sea ice carbon contributions to the Arctic ecosystem: a case study using highly branched isoprenoid lipid biomarkers in Cumberland Sound ringed seals. Limnol. Oceanogr. 59, 1581–1589. (10.4319/lo.2014.59.5.1581)

[43] Brown TA, Chrystal E, Ferguson SH, Yurkowski DJ, Watt CA, Hussey NE, Kelley TC, Belt ST 2017 Coupled changes between the H-Print biomarker and δ**^15^**N indicates a variable sea ice carbon contribution to the diet of Cumberland Sound beluga whales. Limnol. Oceanogr. 62, 1606–1619. (10.1002/lno.10520)

[44] Richard P, Stewart DB 2008 Information relevant to the identification of critical habitat for Cumberland Sound belugas (*Delphinapterus leucas*). *DFO Can. Sci. Advis. Sec. Res. Doc.* 2008/085.

[45] Ulrich KL 2013 Trophic ecology of Arctic char (*Salvelinus alpinus* L.) in the Cumberland Sound region of the Canadian Arctic. Master's Thesis, Department of Biological Sciences, University of Manitoba, Winnipeg, Canada.

[46] Hussey NE et al. 2017 Movements of a deep-water fish: establishing marine fisheries management boundaries in coastal Arctic fisheries. Ecol. Appl. 27, 687–704. (10.1002/eap.1485) 27984681

[47] Spares AD, Stokesbury MJW, Dadswell MJ, O'Dor RK, Dick TA 2015 Residency and movement patterns of Arctic charr *Salvelinus alpinus* relative to major estuaries. J. Fish Biol. 86, 1754–1780. (10.1111/jfb.12683) 25943228

[48] Vander Zanden MJ, Clayton MK, Moody EK, Solomon CT, Weidel BC 2015 Stable isotope turnover and half-life in animal tissues: a literature synthesis. PLoS ONE 10, e0116182. 25635686 10.1371/journal.pone.0116182 PMC4321325

[49] Tomy GT, Budakowski W, Halldorson T, Helm PA, Stern GA, Friesen K, Pepper K, Tittlemeier SA, Fisk AT 2004 Fluorinated organic compounds in an eastern Arctic marine food web. Environ. Sci. Technol. 38, 6475–6481. (10.1021/es049620g) 15669302

[50] Hobson KA, Fisk AT, Karnovsky N, Holst M, Gagnon J-M, Fortier M 2002 A stable isotope (δ **^15^**N and δ **^13^**C) model for the North Water food web: implications for evaluating trophodynamics and the flow of energy and contaminants. Deep-Sea Res. II 49, 5131–5050. (10.1016/S0967-0645(02)00182-0)

[51] Post DM, Layman CA, Arrington DA, Takimoto G, Quattrochi J, Montana CG 2007 Getting to the fat of the matter: models, methods and assumptions for dealing with lipids in stable isotope analyses. Oecologia 152, 179–189. (10.1007/s00442-006-0630-x) 17225157

[52] Yurkowski DJ, Hussey NE, Semeniuk C, Ferguson SH, Fisk AT 2015 Effects of lipid extraction and the utility of lipid normalization models on δ**^13^**C and δ**^15^**N values in Arctic marine mammal tissues. Polar Biol. 38, 131–143. (10.1007/s00300-014-1571-1)

[53] Bligh EG, Dyer WJ 1959 A rapid method of total lipid extraction and purification. Can. J. Biochem. Phys. 37, 911–917. (10.1139/y59-099) 13671378

[54] Parnell A, Jackson A 2013 SIAR: Stable isotope analysis in R. R package version 4.2. See http://CRAN.R-project.org/package=siar (accessed 8 October 2016).

[55] R Development Core Team. 2016 R: A language and environment for statistical computing. Vienna, Austria: R Foundation for Statistical Computing.

[56] Phillips DL, Inger R, Bearhop S, Jackson AL, Moore JW, Parnell AC, Semmens BX, Ward EJ 2014 Best practices for use of stable isotope mixing models in food-web studies. Can. J. Zool. 92, 823–835. (10.1139/cjz-2014-0127)

[57] Hobson KA, Schell DM, Renouf D, Noseworthy E 1996 Stable carbon and nitrogen isotopic fractionation between diet and tissues of captive seals: implications for dietary reconstructions involving marine mammals. Can. J. Fish. Aquat. Sci. 53, 528–533. (10.1139/f95-209)

[58] Caut S, Laran S, Garcia-Hartmann E, Das K 2011 Stable isotopes of captive cetaceans (killer whales and bottlenose dolphins). J. Exp. Biol. 214, 538–545. (10.1242/jeb.045104) 21270301

[59] Caut S, Angulo E, Courchamp F 2009 Variation in discrimination factors (Δ**^15^**N and Δ**^13^**C): the effect of diet isotopic values and applications for diet reconstruction. J. Applied Ecol. 46, 443–453. (10.1111/j.1365-2664.2009.01620.x)

[60] Hussey NE, MacNeil MA, McMeans BC, Olin JA, Dudley SF, Cliff G, Wintner SP, Fennessy ST, Fisk AT 2014 Rescaling the trophic structure of marine food webs. Ecol. Lett. 17, 239–250. (10.1111/ele.12226) 24308860 PMC3912912

[61] Smith JA, Mazumber D, Suthers IA, Taylor MD 2013 To fit or not to fit: evaluating stable isotope mixing models using simulated polygons. Methods Ecol. Evol. 4, 612–618. (10.1111/2041-210X.12048)

[62] Jackson AL, Inger R, Parnell AC, Bearhop S 2011 Comparing isotopic niche widths among and within communities: SIBER–Stable Isotope Bayesian Ellipses in R. J. Anim. Ecol. 80, 595–602. (10.1111/j.1365-2656.2011.01806.x) 21401589

[63] Layman CA, Arrington DA, Montaña CG, Post DM 2007 Can stable isotope ratios provide for community-wide measures of trophic structure? Ecology 88, 42–48. (10.1890/0012-9658(2007)88%5B42:CSIRPF%5D2.0.CO;2) 17489452

[64] Post E et al. 2013 Ecological consequences of sea-ice decline. Science 341, 519–524. (10.1126/science.1235225) 23908231

[65] MacArthur RH, Pianka ER 1966 On optimal use of a patchy environment. Am. Nat. 100, 603–609. (10.1086/282454)

[66] Yurkowski DJ, Hussey NE, Fisk AT, Ferguson SH 2017 Temporal shifts in intraguild predation pressure between beluga whales and Greenland halibut in a changing Arctic. Biol. Lett. 13, 20170433 (10.1098/rsbl.2017.0433) 29118241 PMC5719374

[67] Yurkowski DJ et al. 2016 Influence of sea ice phenology on the movement ecology of ringed seals across their latitudinal range. Mar. Ecol. Prog. Ser. 562, 237–250. (10.3354/meps11950)

[68] Giraldo C, Stasko A, Walkusz W, Majewski A, Rosenberg B, Power M, Swanson H, Reist JD 2018 Feeding of Greenland halibut (*Reinhardtius hippoglossoides*) in the Canadian Beaufort Sea. J. Mar. Sys. 183, 32–41. (10.1016/j.jmarsys.2018.03.009)

[69] Dennard ST, McMeans BC, Fisk AT 2009 Preliminary assessment of Greenland halibut diet in Cumberland Sound using stable isotopes. Polar Biol. 32, 941–945. (10.1007/s00300-009-0624-3)

[70] Rikardsen AH, Amundsen PA 2005 Pelagic marine feeding of Arctic char and sea trout. J. Fish Biol. 66, 1163–1166. (10.1111/j.0022-1112.2005.00655.x)

[71] Harwood LA, Smith TG, George JC, Sandstrom SJ, Walkusz W, Divoky GJ 2015 Change in the Beaufort Sea ecosystem: diverging trends in body condition and/or production in five marine vertebrate species. Prog. Oceanogr. 136, 263–273. (10.1016/j.pocean.2015.05.003)

[72] Wisz MS et al. 2015 Arctic warming will promote Atlantic-Pacific fish interchange. Nat. Clim. Change 5, 261–265. (10.1038/nclimate2500)

[73] Frainer A, Primicerio R, Kortsch S, Aune M, Dolgov AV, Fossheim M, Aschan MM 2017 Climate-driven changes in functional biogeography of Arctic marine fish communities. Proc. Natl Acad. Sci. USA 114, 12 202–12 207. (10.1073/pnas.1706080114) PMC5699037 29087943

[74] Dirzo R, Young HS, Galetti M, Ceballos G, Isaac NJ, Collen B 2014 Defaunation in the Anthropocene. Science 345, 401–406. (10.1126/science.1251817) 25061202

[75] Pinsky ML, Worm B, Fogarty MJ, Sarmiento JL, Levin SA 2013 Marine taxa track local climate velocities. Science 341, 1239–1242. (10.1126/science.1239352) 24031017

