# Supplementary material for: A temporal shift in trophic diversity among a predator assemblage in a warming Arctic
Source: R Soc Open Sci. 2018 Oct 3;5(10):180259. doi: 10.1098/rsos.180259 (PMC6227933; doi:10.1098/rsos.180259)
Supplement: Electronic Supplementary Material 1 [file rsos180259supp1.docx]

**Supplementary Materials**

**Article title:** A temporal shift in trophic diversity among a predator assemblage in a warming Arctic

**Author list:** David J. Yurkowski, Nigel E. Hussey, Steven H. Ferguson and Aaron T. Fisk

**Consideration of assumptions**

Two factors could potentially impact our data interpretation: 1) oceanic Suess effect, and 2) long-term variation in isotopic baselines. When considering the oceanic Suess effect, δ^13^C values for each predator species within both time periods (20 years and 8 years before the present) were not corrected prior to analysis. No correction was undertaken given minimal changes of the Suess effect per decade (-0.18‰) [1] relative to the timeframe of our observations. For example, maximal changes in δ^13^C (<0.4‰) within the early time period (1982-2002) would lead to a negligible change in mixing model results (see [2]). The degree of change during the later time period (2004-2012) is even less (<0.18‰) and slightly above the measurement error for the mass spectrometer (0.1‰). It was therefore deemed unnecessary to correct these data. With regard to variation in isotopic baselines, δ^13^C and δ^15^N values of plankton (i.e. baseline of the food web) have not been investigated over the long-term (i.e. many decades) anywhere in the Arctic, however spatial variability in δ^13^C is linked to temperature and dissolved CO_2_ and for δ^15^N is linked to nitrogen source, N_2_-fixation, denitrification, nitrogen assimilation and nutrient pool size [3]. Visually inspecting the spatial variability of δ^13^C-plankton in waters near Cumberland Sound from isoscape maps developed by [4] and summer sea temperature gradients in the same area (a change of 1-2°C; <https://www.seatemperature.org/>), δ^13^C-plankton remained relatively consistent (~ -22.5‰). Therefore, a 1.3°C change in sea surface temperature within Cumberland Sound over the 30-yr study period would likely have minimal influence on δ^13^C-plankton values. Furthermore, in Igloolik, Nunavut, Canada (69°22′34″N 081°47′58″W), located at a similar latitude, the δ^15^N of phenylalanine of walrus muscle tissue did not significantly change over a 27-yr period (Yurkowski, unpubl. data). Phenylalanine is a source amino acid which does not undergo trophic fractionation, and thus represents the baseline [5]. The walrus population within this area also has a restricted distribution.

**Results of dietary, isotopic niche and community metrics from 2007-2012**

*Stable isotope mixing models:*

Stable isotope mixing model results from 2007-2012 revealed that median contributions to Cumberland Sound beluga whale and ringed seal diet were similar to results from 2005-2012. In the 2007-2012 time period, beluga whale diet consisted of forage fish (43%; 95% Bayesian credible interval (15-70), Greenland halibut (38%; 20-55) and squid (13%; 0-28). Ringed seal diet consisted of squid (50%; 38-63) and forage fish (44%; 26-59). From 2005-2012, beluga whale diet consisted of forage fish (35%; 3-56), Greenland halibut (42%; 27-63) and squid (14%; 0-31). Ringed seal diet was comprised of squid (48%; 39-58) and forage fish (49%; 34-60).

*Isotopic niche size:*

From 2007-2012, the SEA_B_ for beluga whales and ringed seals were 0.69 (0.36-1.18) and 1.21 (0.82-1.71), respectively. Comparably, from 2005-2012 the SEA_B_ for beluga whales and ringed seals were 0.96 (0.63-1.41) and 1.30 (0.98-1.69), respectively.

*Community-wide metrics:*

For the community-wide metrics from 2007-2012, the δ^13^C range was 0.91 (0.33-1.55), the δ^15^N range was 1.98 (1.44-2.48), the mean distance to centroid was 0.94 (0.72-1.16), the mean nearest neighbour distance was 0.68 (0.30-1.06), the standard deviation of nearest neighbour distance was 0.25 (0.00-0.62) and total area was 1.00 (0.30-1.77). Similarly, from 2005-2012, the δ^13^C range was 1.11 (0.62-1.60), the δ^15^N range was 2.01 (1.59-2.46), the mean distance to centroid was 0.98 (0.81-1.15), the mean nearest neighbour distance was 0.67 (0.35-0.97), the standard deviation of nearest neighbour distance was 0.45 (0.07-0.80) and total area was 1.00 (0.46-1.58).

**Table S1.** Sample sizes (n) and collection years of each predator and prey species sampled during the open water period (late May to early November) in Cumberland Sound and western Davis Strait. Note: Arctic cod samples from the 2005-2012 time period were collected in 2004.

| Common name | | | Species name | n | Years collected |
| --- | --- | --- | --- | --- | --- |
| **1990 - 2002** | | |  |  |  |
|  | **Predator** | |  |  |  |
|  |  | Beluga | *Delphinapterus leucas* | 47 | 1992, 1995, 1997, 2000, 2001, 2002 |
|  |  | Ringed seal | *Pusa hispida* | 175 | 1990, 1992, 1993, 1994, 1996, 1999, 2002 |
|  |  | Greenland halibut | *Reinhardtius hippoglossoides* | 14 | 1996 |
|  |  | Arctic char | *Salvelinus alpinus* | 72 | 2002 |
|  | **Prey** | |  |  |  |
|  |  | Squid | *Gonatid sp.* | 7 | 2001 |
|  |  | Shrimp | *Pandalus borealis* | 10 | 2000, 2001 |
|  |  | Arctic cod | *Boreogadus saida* | 8 | 2000, 2001 |
|  |  | Greenland halibut | *Reinhardtius hippoglossoides* | 14 | 1996 |
|  |  |  |  |  |  |
| **2005 - 2012** | | |  |  |  |
|  | **Predator** | |  |  |  |
|  |  | Beluga | *Delphinapterus leucas* | 25 | 2005, 2006, 2007, 2008, 2009 |
|  |  | Ringed seal | *Pusa hispida* | 53 | 2006, 2007, 2008, 2009, 2010, 2011 |
|  |  | Greenland halibut | *Reinhardtius hippoglossoides* | 21 | 2012 |
|  |  | Arctic char | *Salvelinus alpinus* | 162 | 2008, 2011 |
|  | **Prey** | |  |  |  |
|  |  | Squid | *Gonatid sp.* | 5 | 2011 |
|  |  | Shrimp | *Lebbeus polaris* | 7 | 2007, 2008, 2009 |
|  |  | Arctic cod/capelin | *Boreogadus saida / Mallotus villosus* | 22 | 2004, 2007, 2008, 2009, 2011 |
|  |  | Greenland halibut | *Reinhardtius hippoglossoides* | 21 | 2012 |


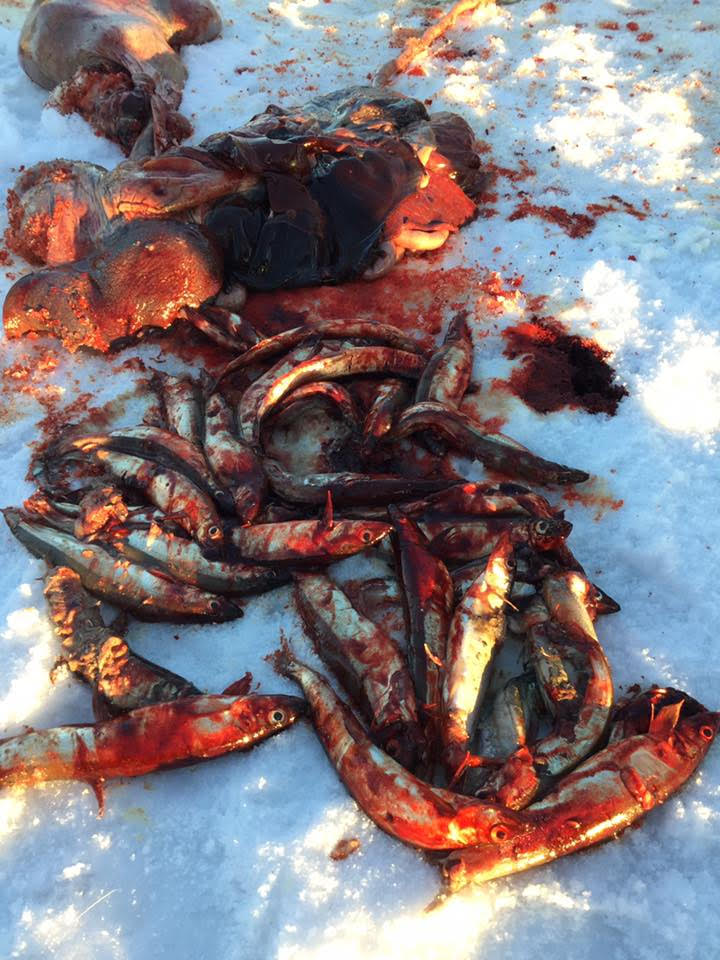


**Figure S1.** Photograph of capelin (*Mallotus villosus*) remains from the stomach contents of a ringed seal (*Pusa hispida*) harvested in near Pangnirtung in Cumberland Sound, Nunavut, Canada in January 2017. Photograph courtesy of Peter Kilabuk.

**
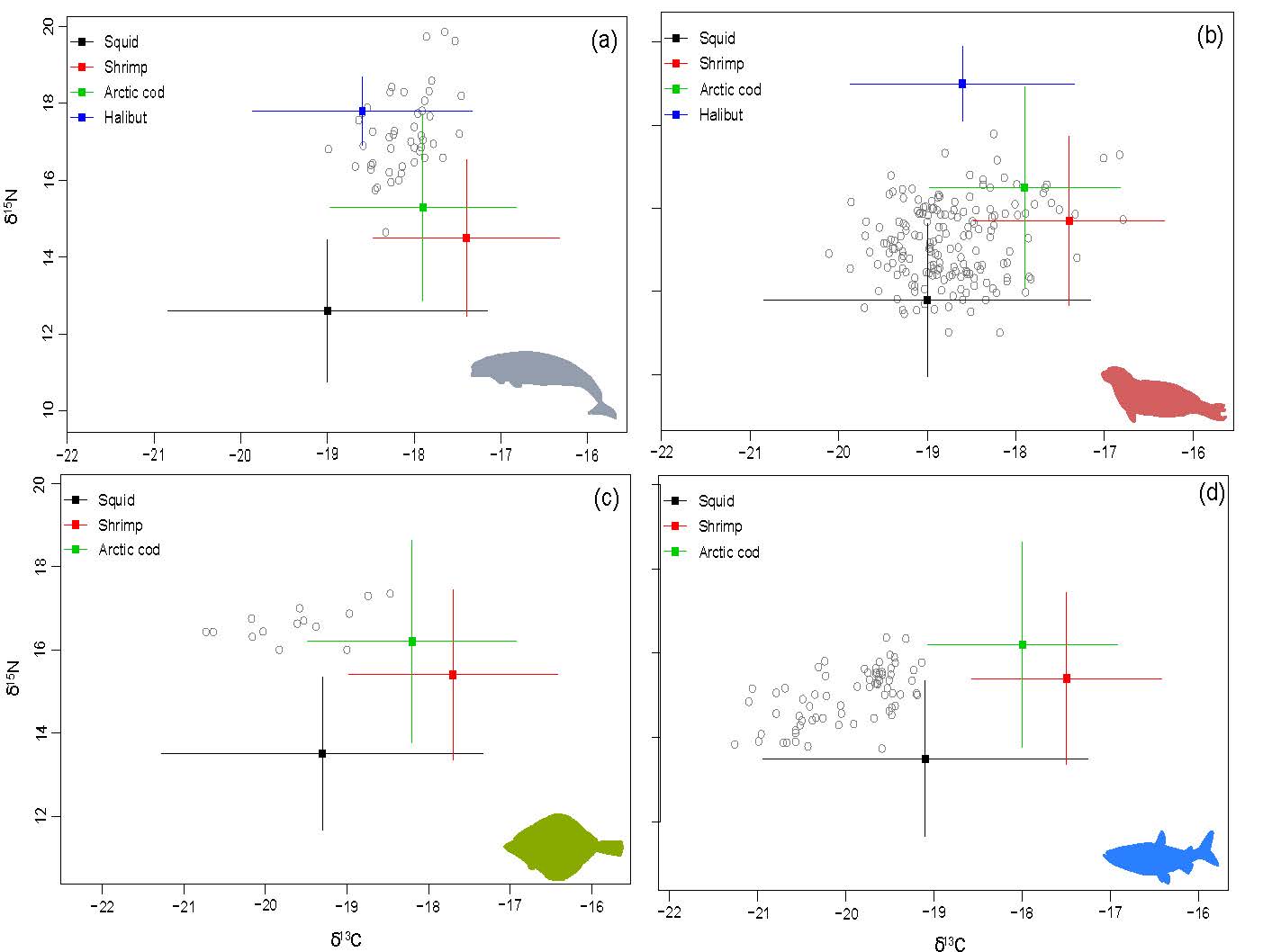
**

**Figure S2.** Stable isotope bi-plot of prey sources from mixing model analysis (mean ± SD) relative to isotopic values of beluga (a), ringed seal (b), Greenland halibut (c) and Arctic char (d) corrected by diet-tissue discrimination factors for the 1990-2002 time period in Cumberland Sound.

**
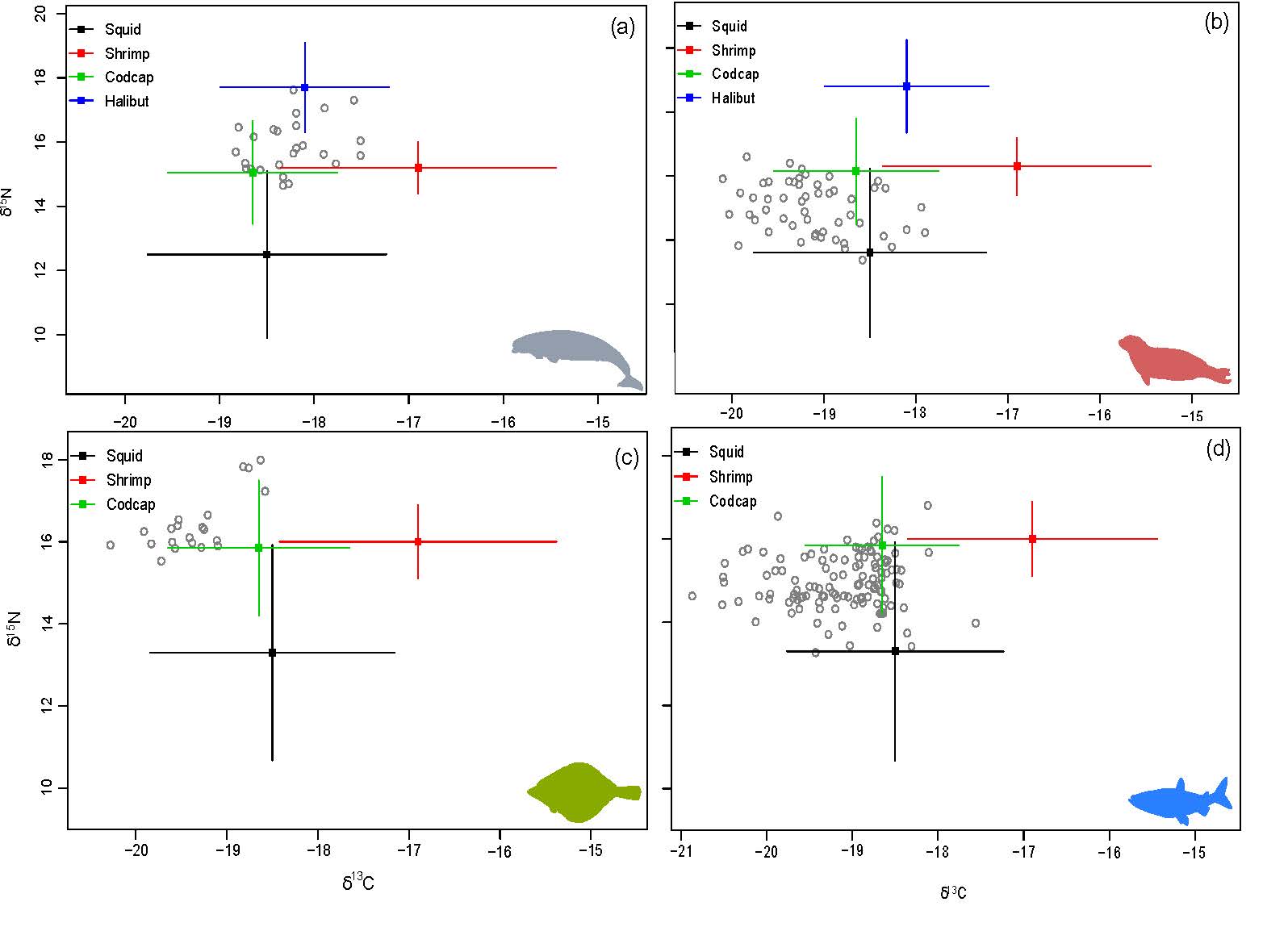
 Figure S3.** Stable isotope bi-plot of prey sources from mixing model analysis (mean ± SD) relative to isotopic values of beluga (a), ringed seal (b), Greenland halibut (c) and Arctic char (d) corrected by diet-tissue discrimination factors for the 2005-2012 time period in Cumberland Sound. Codcap: combined stable isotope values of Arctic cod/Capelin


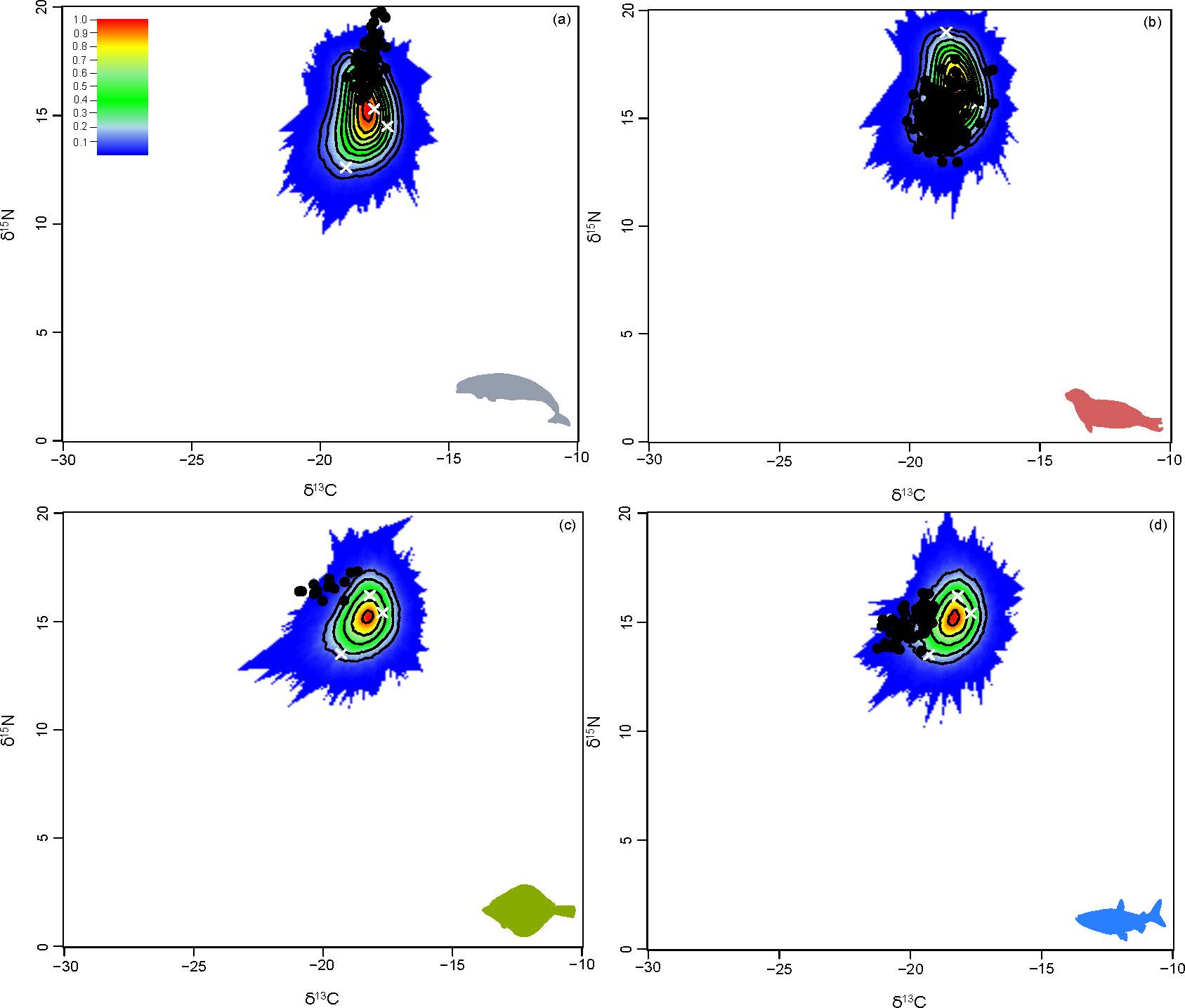


**Figure S4.** Simulated mixing regions from mixing model analysis beluga (a), ringed seal (b), Greenland halibut (c) and Arctic char (d) corrected by diet-tissue discrimination factors for the 1990-2002 time period in Cumberland Sound. Probability contours from the 5% (dark blue) to 100% (red) are shown.


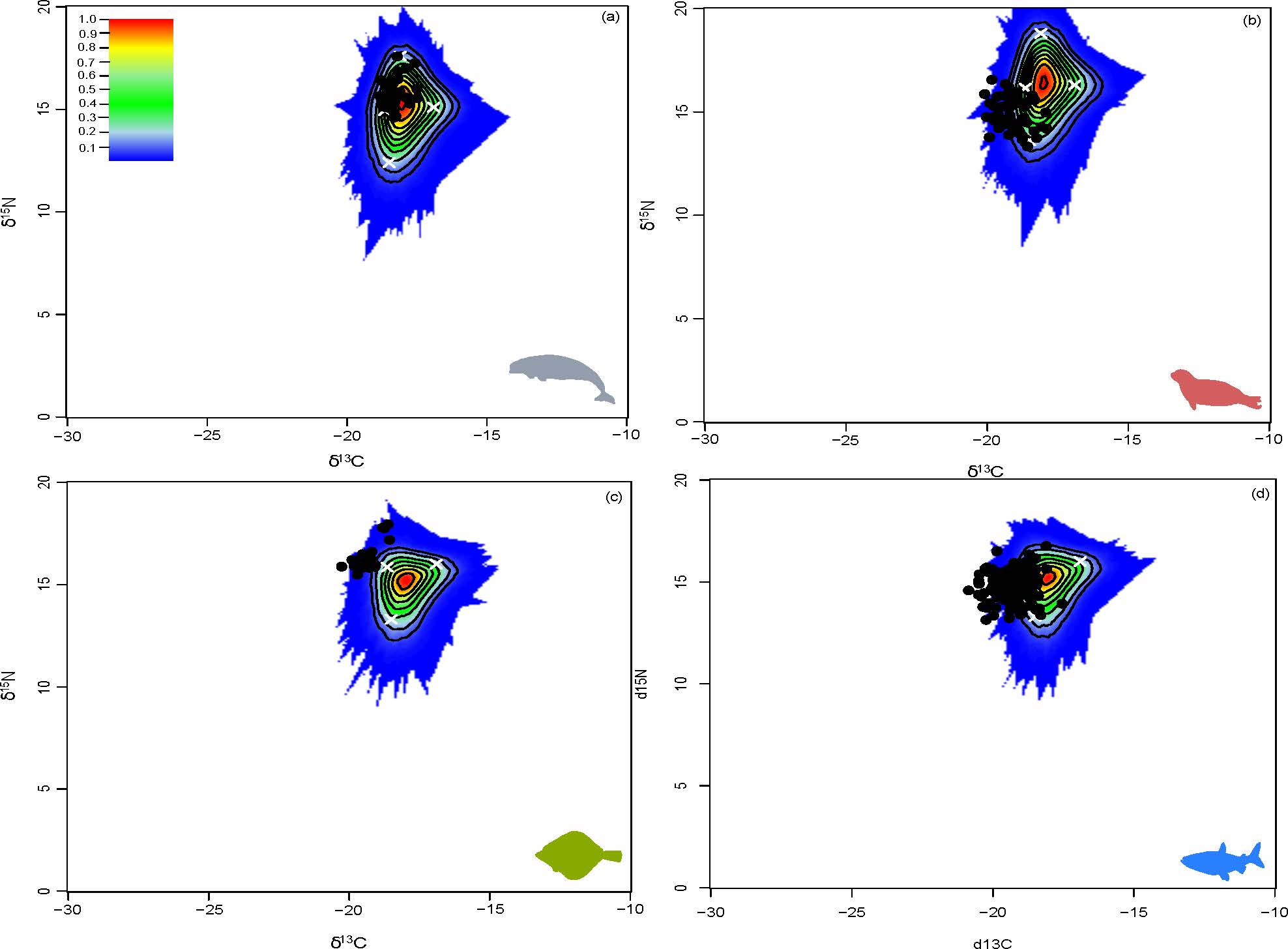


**Figure S5.** Simulated mixing regions from mixing model analysis beluga (a), ringed seal (b), Greenland halibut (c) and Arctic char (d) corrected by diet-tissue discrimination factors for the 2005-2012 time period in Cumberland Sound. Probability contours from the 5% (dark blue) to 100% (red) are shown.

**References**

1. Gruber N, Keeling CD, Bacastow RB, Guenther PR, Lueker TJ, Wahlen M, Meijer HAJ, Mook WG & Stocker TF (1999) Spatiotemporal patterns of carbon-13 in the global surface oceans and the oceanic Suess effect. *Global Biogeochem. Cyc.* **13**, 307–335.
2. Bond AL & Diamond AW (2011) Recent Bayesian stable-isotope mixing models are highly sensitive to variation in discrimination factors. *Ecol. Appl.* **21**, 1017–1023.
3. Graham BS, Koch PL, Newsome SD, McMahon KW & Aurioles D (2010) Using iscoscapes to trace the movements and foraging behaviour of top predators in oceanic ecosystems. *Iscoscapes* 299–318.
4. McMahon KW, Hamady LL & Thorrold SR (2013) A review of ecogeochemistry approaches to estimating movements of marine animals. *Limnol. Oceanogr.* **58**, 697–714.
5. Chikaraishi Y, Kashiyama Y, Ogawa NO, Kitazoto H, Ohkouchi N (2003) Metabolic control of nitrogen isotopes composition of amino acids in macroalgae and gastropods: implications for aquatic food web studies. *Mar. Ecol. Prog. Ser.* **342**, 85–90.
